# Hypofractionation in Prostate Cancer: Radiobiological Basis and Clinical Appliance

**DOI:** 10.1155/2014/781340

**Published:** 2014-04-30

**Authors:** M. Mangoni, I. Desideri, B. Detti, P. Bonomo, D. Greto, F. Paiar, G. Simontacchi, I. Meattini, S. Scoccianti, T. Masoni, C. Ciabatti, A. Turkaj, S. Serni, A. Minervini, M. Gacci, M. Carini, L. Livi

**Affiliations:** ^1^Radiotherapy Unit, Department of Experimental and Clinical Biomedical Sciences, University of Florence, Largo Brambilla 3, 50134 Firenze, Italy; ^2^Urology Unit, Department of Experimental and Clinical Medicine, University of Florence, 50134 Florence, Italy

## Abstract

External beam radiation therapy with conventional fractionation to a total dose of 76–80 Gy represents the most adopted treatment modality for prostate cancer. Dose escalation in this setting has been demonstrated to improve biochemical control with acceptable toxicity using contemporary radiotherapy techniques. Hypofractionated radiotherapy and stereotactic body radiation therapy have gained an increasing interest in recent years and they have the potential to become the standard of care even if long-term data about their efficacy and safety are not well established. Strong radiobiological basis supports the use of high dose for fraction in prostate cancer, due to the demonstrated exceptionally low values of **α**/**β**. Clinical experiences with hypofractionated and stereotactic radiotherapy (with an adequate biologically equivalent dose) demonstrated good tolerance, a PSA control comparable to conventional fractionation, and the advantage of shorter time period of treatment. This paper reviews the radiobiological findings that have led to the increasing use of hypofractionation in the management of prostate cancer and briefly analyzes the clinical experience in this setting.

## 1. Introduction

Prostate cancer represents the most common male cancer diagnosed in Western countries after nonmelanomatous skin cancer [1]. Since in most cases the prostate cancer at diagnosis is organ confined [2], radical prostatectomy and definitive radiotherapy are the accepted standard for treating the vast majority of prostate cancer cases. External beam radiation therapy (EBRT) is the most diffused radiotherapy treatment modality for treating patients with prostate cancer. A conformal delivery of treatment is ideally adopted in order to spare as much as possible the amount of radiation received by the surrounding normal tissues. Nowadays, conventionally fractionated radiation therapy (CFRT, a single 1.8–2.0 Gy fraction lasting one hour per day, five days per week, for about eight weeks) to a total dose of 76–80 Gy represents the most adopted treatment modality. Dose escalation in this setting has been demonstrated to improve biochemical control with acceptable toxicity using contemporary radiotherapy techniques [3, 4]. CFRT schemes employing fraction sizes of 1.8–2.0 Gy are based upon the premise that tumors typically are less responsive to fraction size than are late responding normal tissues. The *α*/*β* ratio is a measure of fractionation response, with low ratios (high *α*/*β*'s) associated with late responding normal tissues. A low *α*/*β* is consistent with a greater capacity for repair between fractions, with an accompanying greater relative sparing with small fraction sizes, than for tumors with their typically higher *α*/*β* ratios. Under these conditions, an improved therapeutic ratio is achieved with multiple small fractions for most types of tumors. The *α*/*β* ratios are thought to be associated with tumors; however, they are typically 8 or greater, whereas for late responding normal tissues, values on the order of 3 or 4 or somewhat less are suggested from the analyses of numerous experimental and some clinical outcome studies. There appear to be exceptions to such typical tumor response to fractionation, however. Growth fraction (or effective cell cycle time) has often been associated with fractionation response, with slowly proliferating normal tissues (and some slowly proliferating tumors) generally displaying stronger than expected fraction size responses (low *α*/*β* ratios).

Hypofractionated radiotherapy (HFRT, a single 2.1–3.5 Gy fraction, five days per week, for around four weeks) has gained a considerable interest in recent years. Stereotactic body radiation therapy (SBRT, a single 3.5–15.0 Gy fraction, five days per week, for about two weeks) has been recently an object of increasing interest in the scientific community due to the technical improvements that have made possible the delivery of larger radiation fraction size; thus, it has the potential to become the standard of care even if long-term data about its efficacy and safety are not well established.

The aim of this paper is to review the radiobiological findings that have led to the increasing use of hypofractionation in the management of prostate cancer and briefly analyze the clinical experience in this setting.

## 2. Radiobiological Basis of Hypofractionation

**Figure 1:**
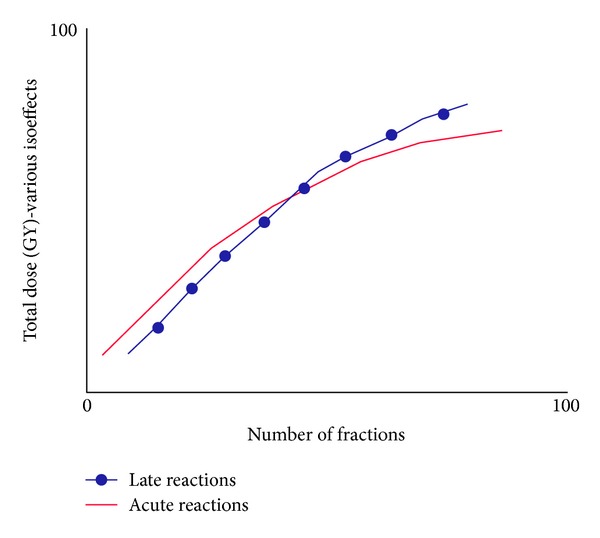
(Based on data from Withers and Thames [5, 6]) the steepness and curvature of these lines are determined by the *α*/*β* ratio. The graph indicates a greater sensitivity of late responses to changes in dose per fraction: using lower doses per fraction tends to spare late reactions.

Considerable efforts are being devoted at the present time to the improvement of radiotherapy and there is no doubt that radiobiology has been very fruitful in the generation of new ideas and in the identification of potentially exploitable mechanisms. In the last years improvements in biological knowledge have changed several aspects of radiobiology. About 40 years ago, Thames and Withers largely studied the influence of dose per fraction on response. In each study and for each chosen dose per fraction the total radiation dose (isoeffective dose) that produced some defined level of damage to the normal tissue or to the tumor was determined [5, 6]. The relationships between total dose and dose per fraction for acutely responding tissues (i.e., high-turnover tissues), late responding tissues (low or no turnover), and tumours provided the basic information required to optimize radiotherapy according to the dose per fraction and number of fractions. Those pioneering studies showed that the isoeffective total dose increased more rapidly with decreasing dose per fraction for late effect than for acute effect, which indicates a greater sensitivity of late responses to changes in dose per fraction (Figure 1). The relationship between total isoeffective dose and the dose per fraction in fractionated radiotherapy can be described using the linear-quadratic (LQ) cell survival model [LQ: ln⁡*S* = *αd* − *βd* ^2^] [7], that is, the standard model for calculating isoeffects in the range of conventional dose per fraction [8]. The steepness and curvature of isoeffective lines are determined by *α*/*β* ratio. At the present time it is strongly recommended that the LQ model should always be used, with a correctly chosen *α*/*β* ratio, to describe isoeffect dose relationships at least over the range of doses per fraction between 1 and 5 Gy [9–11]. The renewed interest for hypofractionation has raised the problem of the need to adapt LQ model to higher dose per fraction because clinically the LQ model often underestimates tumor control observed at radiosurgical doses [12]. However, recent papers conclude that the available data do not support a need to change the LQ model at large dose per fraction, if *α*/*β* ratio is selected appropriately [8, 13]. A possible explanation for the difference in shape of dose-response relationships for early and late responding tissues is the different distribution of the cells through the cycle. The radiosensitivity of a population of cells varies with the distribution of cells through the cycle, with a greater radioresistance in the late phase *S*, in the early *G*_1_, and in the quiescence phase *G*_0_ [14]; likely many late responding normal tissues are resistant owing to the presence of many not-proliferating cells that are resting in *G*_0_. Early responding tissues that proliferate quickly can have a part of cell in a radioresistant phase, but the redistribution through all the phases of the cell cycle allows the cells to be in more sensitive phases at the next fraction of radiation. At the same time, the fast proliferation itself is a form of resistance that increases the total number of cells to kill. Repopulation occurring during a protracted, fractionated regimen helps to spare normal tissues but is a potential danger for the control of tumor. If the overall duration of fractionated radiotherapy is increased, there will usually be greater repopulation of the irradiated tissues, both in the tumor and in early-reacting normal tissues. To counteract proliferation of tumor cells, an extra dose is needed. The proliferation is relevant in mouse skin about 2 weeks after the start of daily fractionation. The longer cell cycle of human cells makes proliferation evident after a longer period [15, 16]. As overall time increases, a greater total dose had been required to control tumors that show an accelerated repopulation of clonogenic tumor cells at some point during fractionated radiotherapy. In head and neck cancers, for treatment times longer than 4 weeks, the effect of proliferation is equivalent to a loss of radiation dose of about 0,48–0.6 Gy/day [17, 18]. Prolonging overall time within the normal radiotherapy range has a large sparing effect on early reactions but little sparing effect on late reactions, because the time at which extra dose is required to counteract proliferation in late responding tissues in humans is far beyond the overall time of any normal radiotherapy regimen [19]. Thus, in acute responding tissues fraction size and overall treatment time both determine the effect; instead for late responding tissues fraction size is the dominant factor in determining the radiation-induced effect. Acute and late responding tissues are usually differentiated on the basis of different alpha beta value, with high alpha beta (in the range of 7–20 Gy) for acute responding tissues and low alpha beta, generally in the range of 0.5–6 Gy, for late responding tissues. It is common practice to apply to tumors the same alpha beta of acute responding tissues of approximately 10 Gy [20, 21]. However there is evidence that some human tumor types exhibit low alpha beta ratios and also breast and early stage prostate cancer [22, 23], perhaps with alpha beta ratios even lower than for late normal tissue reaction. This can be due not only to different tumor characteristics but also to cell variability into the tumor or uncontrolled confounding factors, such as the presence of tumor hypoxia, repopulation, or patient-to-patient variability.

As Withers wrote in 1985 “conventional is commonly not universally correct, and so with dose fractionation in radiotherapy” [5]. The heterogeneity of cancer cells and cancer types and the importance of overall treatment time make altered fractionation more useful in selected cases.

## 3. Brief History of Radiobiological Hypothesis Concerning **α**/**β** of Prostate Cancer

In radiobiology, the *α*/*β* ratio is used to estimate the effects of radiation on various tissues and compare various dose and fractionation schemes. The *α*/*β* ratio is estimated to be >10 Gy for early-responding tissues (e.g., skin, mucosa, and most tumors) and 3–5 Gy for late responding tissues (e.g., connective tissue, bladder/rectal mucosa, and muscles). In 1999 Brenner and Hall [10] promoted the hypothesis that prostate tumors have exceptionally low values of *α*/*β*. They derived an *α*/*β* ratio of 1.5 Gy with a 95% confidence interval of 0.8 to 2.2 Gy, based on 367 patients from two treatment centers. Their assumption stems from the documented result that similar biochemical long-term control is achieved using EBRT doses of about 70 Gy in 1.8–2.0 Gy fractions but using 145 Gy from permanent iodine-125 (I125) low-dose-rate (LDR) irradiation. In 2001, Fowler et al. [24] updated this analysis with 1020 patients from 11 centers and came to the same result of *α*/*β* = 1.5 Gy, with a narrower confidence interval (1.25–1.75 Gy). These ranges of values were confirmed in a 2012 paper by Miralbell et al. [25] where a retrospective study was performed on nearly 6,000 prostate cancer patients from seven international institutional primary datasets treated with EBRT stratified by risk groups and androgen deprivation status. A direct analysis of 5-year biochemical relapse–free survival (bRFS) data with the linear-quadratic (LQ) model was implemented to estimate the dose fractionation sensitivity for this group of patients. Since the initial hypothesis about a low *α*/*β* for prostate cancer derived from brachytherapy data, a specific concern was expressed toward the fact that neither Brenner and Hall [10] nor Fowler et al. [24] assumed that repopulation in the tumors was significant during low-dose-rate treatment with I125. Wang et al. [26] and Kal and van Gellekom [27] took in account the effect of tumor repopulation in their work to derive LQ parameters for prostate cancer: all authors postulated that this effect is not negligible for the accurate description of the radiation therapy of prostate. This consideration caused a 23% reduction of I125 dose from 145 to 112 Gy and resulted in an estimate of *α*/*β* = 3-4 Gy instead of the previously derived 1.5 Gy. Furthermore, the work of Brenner and Hall [10] was questioned by King and Mayo [28] because of its extremely low radiosensitivity (*α* = 0.036 Gy^−1^). Such a low *α* value leads to excessively low clonogenic cell numbers (in the range of 10 to 100); the authors proposed that a solid tumor would consist of a heterogeneous population of clonogens with a spectrum of radiosensitivities. Recently, Pedicini et al. [29] proposed a method to estimate intrinsic radiosensitivity (*α*), fractionation sensitivity (*α*/*β*), repopulation doubling time, number of clonogens, and kick-off time for accelerated repopulation of prostate cancer. They confirmed a low value of *α*/*β*, 2.96 Gy (95% CI 2.41–3.53 Gy), with a correspondingly high value of intrinsic radiosensitivity, 0.16 Gy^−1^ (95% CI 0.14–0.18 Gy^−1^), a realistic average number of clonogens, a long kick-off time for accelerated repopulation, and a surprisingly fast repopulation that suggests the involvement of subpopulations of specifically tumorigenic stem cells during continuing radiation therapy.

Finally it should be noted that all the above calculations agreed on a small value of *α*/*β*, providing an attracting rationale to utilize HFRT in prostate cancer.

## 4. Clinical Experiences Involving HFRT

Zaorsky et al. [30] recently published an extensive review concerning the history of HFRT. As stated by the authors, the first initial retrospective experience reported about the use of HFRT came from the UK. Over 200 patients were treated at St. Thomas Hospital in London with hypofractionated radiotherapy to a dose of 55 Gy in 12 fractions and later to doses of 36 Gy in 6 fractions with low rectal and urological complications [31, 32]. The trial included men with early (T1-T2) and advanced (T3-T4) disease that were treated by external beam radiotherapy. Depending on anatomy, patients were treated with 3-field, 4-field, or a double rotation technique from a cobalt-60 machine or linear accelerator.

One of the first phase III prospective randomized controlled trials (RCTs) comparing CFRT and HFRT was published by Lukka et al. [33] in 2005: in this trial a conventional dose of 66 Gy in 33 fractions was compared to a hypofractionated regimen of 52.5 Gy in 20 fractions (dose per fraction = 2.625 Gy) in more than 900 men with low and intermediate risk prostate cancer. Surprisingly, the 5-year rate of failure (both biochemical and clinical) was higher in the hypofractionated arm compared to the standard fractionation arm (60% versus 53%, *P* < 0.05). The worse outcome reported in the hypofractionated arm may be explained by the fact that, for any *α*/*β* ratio >0.2, the biologically equivalent dose (BED) of 52.5 Gy in 20 fractions is expected to be lower than the BED of 66 Gy in 33 fractions. At a median followup of 5.7 years, no difference in 5-year actuarial rate of late grade 3 or higher gastrointestinal or genitourinary toxicity was observed between the two arms. Subsequently, Yeoh et al. [34, 35] reported that opposite results regarding 217 patients with T1-2 prostate carcinomas were randomized to either a CFRT or a HFRT arm between 1996 and 2006. Treatments were predominantly four-field box technique with customized blocks using 6–23 MV photons. Patient in the CFRT arm received a modest dose of 64 Gy in 32 fractions, while patients in the HFRT arm received a total dose of 55 Gy in 20 treatments. The study population was represented by men with favorable-risk prostate cancer. At a median followup of 90 months, biochemical relapse-free survival (bRFS) was significantly better with hypofractionation when Phoenix definition was used (53% versus 34%, *P* < 0.5). The contrary results reported by these two studies may be caused by a different number of reasons: first, no specific assumptions about the *α*/*β* ratio before the beginning of both trials. Second, the total dose of the CFRT arms was 66 Gy and 64 Gy, respectively, which is considerably lower than more contemporary conventional doses of 78–80 Gy utilized nowadays [3]. Finally, a different definition of biochemical failure (BF) was utilized in the two studies: while the Lukka et al. study [33] used mainly the ASTRO definition [18] (3 consecutive PSA rises), Yeoh et al.'s study [34, 35] used the ASTRO and Phoenix [36, 37] (nadir + 2 ng/mL) definitions.

After the publication of these initial RCTs comparing CFRT and HFRT, modern prospective phase III superiority trials were initiated based on the assumption that the *α*/*β* ratio for prostate cancer is 1.5 Gy. Dose escalation studies [38–45] have been utilized to determine the standard of care in determining the optimal CFRT schedule. Dearnaley et al. [38] conducted a pilot for a phase III trial randomising 64 Gy versus 74 Gy and reported 5-year biochemical control rates of 59% (standard dose) and 71% (escalated dose) (hazard ratio 0.64, 95% CI 0.38–1.10, *P* = 0.10) with acceptable acute and late toxicity [27]. The subsequent MRC RT01 trial [39] randomised 862 men to the same fractionation regimens and found that at 6 months after radiotherapy grade 2 or higher toxicity was low [28]. However almost all of this toxicity was seen in the group receiving 74 Gy. In both arms the radiotherapy was given in conjunction with androgen deprivation. This trial did also confirm an increase in biochemical progression-free survival (60% with the lower dose and 71% with the higher dose at 5-year followup, hazard ratio of 0.67 for clinical progression in the higher dose arm, CI 0.53–0.85, *P* = 0.0007) and metastasis-free survival, in addition to a reduction in need for salvage androgen suppression. Kupelian et al. [40] pooled the data from nine institutions totaling over 4800 men. Despite the higher dose cohort (>72 Gy) having worse prognostic features, their 5-year biochemical disease-free survival (bDFS) was significantly improved compared to the cohort who received <72 Gy [29]. Pollack et al. [41] conducted a phase 3 trial comparing 70 Gy to 78 Gy without androgen deprivation and found a significant improvement in freedom from failure (including biochemical failure) in the higher dose group (freedom from failure at 6 years: 64% versus 70%, *P* = 0.03 [30]. This included a reduction in the incidence of distant metastasis in the subgroup of patients with a PSA >10 ng/mL at 6 years of followup. However this trial also confirmed an increase in rectal side effects in the higher dose arm (grade 2 or higher toxicity: 26% versus 12%). This trial was conducted in the era before image-guided radiotherapy (IGRT) and intensity-modulated radiotherapy (IMRT) were standard and hence higher doses are likely to be deliverable with less toxicity today. Peeters et al. [42] also conducted a dose escalation trial randomizing 664 men 68 Gy or 78 Gy. The higher dose was associated with a 10% increase in freedom from failure at 5 years (HR 0.74, *P* = 0.02) [31]. Zelefsky et al. [43] reported their experience of treating over 2000 men between 1998 and 2004 in a nonrandomised observational study. They found that increasing dose was associated with better disease control for intermediate and high-risk patients but did not find a statistically significant association with the low-risk patients. Most patients in this study received neoadjuvant hormone therapy.

Arcangeli et al. [44–46] compared HFRT versus CFRT in patients with high-risk prostate cancer. The purpose of this study was to compare the toxicity and efficacy of hypofractionated (62 Gy/20 fractions/5 weeks, 4 fractions per week) versus conventional fractionation radiotherapy (80 Gy/40 fractions/8 weeks). From January 2003 to December 2007, 168 patients were randomized to receive either hypofractionated or conventional fractionated schedules of three-dimensional conformal radiotherapy to the prostate and seminal vesicles. All patients received a 9-month course of total androgen deprivation. There was no reported difference in late toxicity at five years between the two schedules. The 3-year freedom from biochemical failure (FFBF) rates was 87% and 79% in the hypofractionation and conventional fractionation groups, respectively (*P* = 0.035). The authors concluded that, with equivalent late toxicity between the two treatment groups, the hypofractionated treatment resulted in better PSA control. Kuban et al. [47] reported on the preliminary outcome and toxicity of a phase III RCT which based the treatment regimens on maintaining equivalent acute toxicities while delivering a higher BED to the prostate. They randomized 102 men to receive CFRT (BED at *α*/*β* of 3 = 121) to a dose of 75.6 Gy in 42 fractions and 102 men to receive HFRT (BED at *α*/*β* of 3 = 130) to a dose of 72 Gy in 30 fractions. The 5-year Phoenix FFBF rates were 92% and 96% (not statistically significant), respectively, and no patient had a clinical failure. Finally, Pollack et al. [48] recently reported the results of the RCT they conducted. Between June 2002 and May 2006, men with favorable- to high-risk prostate cancer were randomly allocated to receive 76 Gy in 38 fractions at 2.0 Gy per fraction (CFRT) versus 70.2 Gy in 26 fractions at 2.7 Gy per fraction (HFRT). High-risk patients received long-term androgen deprivation therapy (ADT), and some intermediate-risk patients received short-term ADT. There were no statistically significant differences in late toxicity between the arms; however, in subgroup analysis, patients with compromised urinary function before enrollment had significantly worse urinary function after HFRT. No differences were observed in the two arms in terms of BF or any other type of failure. The authors concluded that even if HFRT did not result in a significant reduction in any type of failure (biochemical and clinical) it is delivered in 2.5 fewer weeks. Men with compromised urinary function before treatment may not be ideal candidates for HFRT. Other RCTs are currently ongoing. RTOG 0415 is a phase III RCT with fractionation schedules similar to the regimen of phase I/II trial by Kupelian et al. [49]. If the a/b ratio for prostate cancer is closer to 10, the trial will demonstrate equivalence between the fractionation regimens; if it is closer to 1.5, the HFRT schedule should produce better rates of biochemical control. While phase I and phase II portions of the CHHiP trial [50] have estimated toxicity, the UK Medical Research Council (MRC) phase III noninferiority study will include over 3000 patients in a 3-arm design to extrapolate the isoeffective dose for complications and address whether HFRT is equivalent to CFRT. The NCIC trial is a noninferiority trial that compares 78 Gy in 2 Gy fractions to 60 Gy in 3 Gy fractions. Its goal is to demonstrate the safety and efficacy of HFRT and evaluate it as a replacement for CFRT.

HDR brachytherapy has been historically used as a form of hypofractionation for treating men affected by prostate cancer. Using this technique, fractionation regimens of 48 Gy in 8 fractions or 54 Gy in 9 fractions over 5 days have demonstrated 70% PSA failure-free survival at 5 years, despite the majority of these patients having high-risk disease [51]. Relapse-free survival at 3 years was 100% for the low-risk patients included in this study. Five percent of patients had grade 3 acute GU toxicity and 21% had grade 2 acute GU toxicity. With regard to late toxicity, one patient had a grade 3 GI toxicity and 11% had grade 2 GU toxicity. Yoshioka et al. updated their results in 2010 and had treated 112 men with 54 Gy in 9 fractions with HDR brachytherapy [52]. The majority of these patients had high-risk disease and also received androgen deprivation therapy (ADT). Overall 5-year bRFS was 83%. This was achieved with 5% acute and 3% late grade 3 toxicity. Another cohort of 117 consecutive patients was treated with escalating doses of 6 fractions HDR from 36 Gy to 43.5 Gy, delivered in 2 insertions one week apart [53]. They report excellent 8-year bRFS of 94% for this group of low- and intermediate-risk prostate cancer patients. Four (3%) patients had grade 3 late urinary toxicity. Recently Demanes et al. have described their experience of treating 298 men with mostly low- and low-intermediate-risk prostate cancer [54]. Approximately half were treated with 36 Gy in six Gy fractions, and the others received 4 fractions of 9.5 Gy over 2 days. The 8-year bRFS was 97%. The grade 3 GU toxicity was 5% overall, 24% grade 2, but this was scored per event, not per patient, and hence the same patient with more than one symptom would be scored multiple times. Late GI toxicity was <1%. Mount Vernon Hospital has published outcomes for a group of men, some with locally advanced prostate cancer [55]. This was a dose escalation study so the first cohort received 34 Gy in 4 fractions over 3 days, the second cohort 36 Gy, and the third cohort 31.5 Gy in 3 fractions over 2 days. Only 25–31% patients had grade 1 or more toxicity at six months and two patients had grade 3 toxicity.

## 5. Clinical Experiences Involving the Use of SBRT for the Treatment of Prostate Cancer

SBRT in the management of prostate cancer constitutes a relatively new option. So far, only phases I-II studies have been published regarding its use, even if phase III trials are currently ongoing [56]. The first prospective trial of SBRT for prostate cancer was published by Madsen et al. [57], who treated 40 patients with SBRT using a daily dose of 6.7 Gy to a total dose of 33.5 Gy (6.7 Gy for 5 fractions). The fractionation schedule was calculated to be equivalent to 78 Gy in 2 Gy fractions using an estimated *α*/*β* ratio of 1.5. At the median followup of 41 months, there were no instances of grade 3 gastrointestinal toxicity and only a single episode of acute grade 3 genitourinary toxicity. There was no grade 3 or higher late toxicities. The PSA control rate was 90% by the Phoenix definition [37]. Tang et al. [58, 59] treated 30 men in a phase I/II study. The eligible men had low-risk prostate cancer and received 5 weekly doses of 7 Gy to a total dose of 35 Gy. The SBRT technique consisted of intensity-modulated radiotherapy (IMRT) with daily image guidance using implanted gold fiducials. All patients had at least 6 months of followup. The treatments were well tolerated and there was no grade 3 or 4 GI/GU toxicity. Although there were initial grade 2 toxicities (13% GU and 7% GI), these scores returned to or improved over baseline at 6 months. The biochemical control after 18 months was 100%. Katz et al. [60, 61] reported an experience of SBRT treatment given to 304 patients with clinically localized prostate cancer. Most received 5 fractions of 7.25 Gy (total dose 36.25 Gy). At a median followup of 40 months (range of 9–58 months), 10 patients died of other causes and 9 were lost to followup. The 4-year actuarial freedom from biochemical failure is 98.5%, 93.0%, and 75%, for the low-, intermediate-, and high-risk groups. Late toxicity included 4.2% RTOG grade 2 GI toxicity, 7.8% GU toxicity and 1.4% grade 3 GU toxicity. Freeman and King [62] report their experience of treating 41 low-risk prostate cancer patients with 35–36.25 Gy in 5 fractions [9]. None received adjuvant hormonal therapy. At a median followup of 5 years the biochemical relapse-free survival was 93%. There has been no grade 3 or higher rectal toxicity. One patient experienced grade 3 GU toxicity after repeated instrumentation. 32% and 16% experienced grade 1-2 late GU and GI toxicity, respectively. King et al. have also published their experience of treating 67 men with low-risk prostate cancer [63]. Patients were treated to a dose of 36.25 Gy in 5 fractions using CyberKnife and median followup was 2.7 years. The 4-year bRFS was 94%. Importantly, they clearly showed that alternate day treatment significantly reduces the chance of GU and GI toxicity compared with daily hypofractionated regimens and recommend that this should be the regimen of choice. Towsend et al. [64] reported an analysis of the first 50 patients treated with CyberKnife radiotherapy for prostate cancer. Most patients were affected with early to intermediate stage prostate cancer. Two patients had metastatic disease at presentation and were excluded. A total of 37 patients received irradiation at a dose of 35 to 37.5 Gy in 5 fractions of 7 to 7.5 Gy per fraction. Assuming an alpha/beta ratio of 1.5 Gy, this process delivered an equivalent dose of 85 to 96 Gy in 2 Gy fractions (EQD2). A subset of patients (*n* = 11) received standard linear accelerator-based pelvic radiation treatment either by intensity-modulated radiation therapy or by tomotherapy and received a boost via the CyberKnife at a dose of 17.6 to 25 Gy in 2 to 5 fractions (EQD2 = 46.6–72 Gy). The mean pretreatment prostate specific antigen and Gleason scores were 9.16 ng/mL and 7, respectively. Grade 2 acute genitourinary toxicity was reported by 10% of patients (*n* = 5). Only 3 patients reported grade 3 acute genitourinary toxicity. No gastrointestinal grade 2 or grade 3 toxicities were reported.  Table 1 summarizes the reported efficacy of SBRT in the abovementioned papers.

So far the results of SBRT studies are very encouraging and stress the potential of SBRT in the management of certain patients with prostate cancer. However all the toxicity data gathered so far come from single-centers experience and are often compared with older radiotherapy technique that did not use state-of-the-art technology such as IMRT or image-guided radiotherapy (IGRT). More solid evidences will be available with the currently ongoing phase III trials (NCT01584258—PACE Study, ISRCTN45905321—Scandinavian HYPO).

## 6. Conclusions

Nowadays EBRT constitutes an established treatment modality for almost all prostate cancer patients. Determining the optimal fractionation scheme has been one of the goals of radiation oncologists. Most of the evidence provided in the last decade by specialized literature about prostate cancer radiosensitivity supports the hypothesis that prostate tumor has an extremely low *α*/*β* ratio, thus encouraging the adoption of hypofractionated schedules in this setting. There is a growing body of compelling evidence supporting the safety and efficacy of abbreviated radiotherapy schedules for prostate cancer. So far results of RCTs comparing HFRT and CFRT have been puzzling due to a different number of factors (different doses, radiation techniques, and contouring policies). Results obtained from short-term SBRT have been promising so far, but longer followup and phase III trials are warranted.

## Figures and Tables

**Table 1:** SBRT efficacy in selected experiences.

Study	Fractionation	Stage	Low risk	High risk	5 yr bRFS
Townsend et al. 2011 [64] 48 patients (37 monotherapy, 11 boost)	35–37.5 Gy in 5 fractions (boost 17.6–25 Gy in 2–5 fractions)	69% T1			Not reported

King et al. 2012 [63] 67 patients	36.25 Gy in 5 fractions	T1c or T2a/b	100%	None	4-year bRFS 94%

Freeman and King 2011 [62] 41 patients	35–36.35 Gy in 5 fractions	Low risk		None	93%

Katz et al. 2010 [61] 304 patients	35–36.25 Gy in 5 fractions	92% T1c	70%	4%	1.3% failed so far (17–30 month FU)

Madsen et al. 2007 [57] 40 patients	33.5 Gy in 5 fractions	T1c or T2a	100%	None	48 month bRFS 90%

Tang et al. 2008 [58] 124 patients	33.5 Gy in 5 fractions	T1c or T2a	100%	None	2 years: 90%

Abbreviations: bRFS: biochemical relapse-free survival.
